# Crude oil impairs immune function and increases susceptibility to pathogenic bacteria in southern flounder

**DOI:** 10.1371/journal.pone.0176559

**Published:** 2017-05-02

**Authors:** Keith M. Bayha, Natalie Ortell, Caitlin N. Ryan, Kimberly J. Griffitt, Michelle Krasnec, Johnny Sena, Thiruvarangan Ramaraj, Ryan Takeshita, Gregory D. Mayer, Faye Schilkey, Robert J. Griffitt

**Affiliations:** 1 Gulf Coast Research Laboratory, School of Ocean Science and Technology, University of Southern Mississippi, Ocean Springs, Mississippi, United States of America; 2 Department of Environmental Toxicology, Texas Tech University, Lubbock, Texas, United States of America; 3 Abt Associates, Suite 201, Boulder, Colorado, United States of America; 4 National Center for Genome Resources, 2935 Rodeo Park Dr E, Santa Fe, NM, United States of America; Northwest Fisheries Science Center, UNITED STATES

## Abstract

Exposure to crude oil or its individual constituents can have detrimental impacts on fish species, including impairment of the immune response. Increased observations of skin lesions in northern Gulf of Mexico fish during the 2010 Deepwater Horizon oil spill indicated the possibility of oil-induced immunocompromisation resulting in bacterial or viral infection. This study used a full factorial design of oil exposure and bacterial challenge to examine how oil exposure impairs southern flounder (*Paralichthys lethostigma*) immune function and increases susceptibility to the bacteria *Vibrio anguillarum*, a causative agent of vibriosis. Fish exposed to oil prior to bacterial challenge exhibited 94.4% mortality within 48 hours of bacterial exposure. Flounder challenged with *V*. *anguillarum* without prior oil exposure had <10% mortality. Exposure resulted in taxonomically distinct gill and intestine bacterial communities. Mortality strongly correlated with *V*. *anguillarum* levels, where it comprised a significantly higher percentage of the microbiome in Oil/Pathogen challenged fish and was nearly non-existent in the No Oil/Pathogen challenged fish bacterial community. Elevated *V*. *anguillarum* levels were a direct result of oil exposure-induced immunosuppression. Oil-exposure reduced expression of immunoglobulin M, the major systemic fish antibody, and resulted in an overall downregulation in transcriptome response, particularly in genes related to immune function, response to stimulus and hemostasis. Ultimately, sediment-borne oil exposure impairs immune function, leading to increased incidences of bacterial infections. This type of sediment-borne exposure may result in long-term marine ecosystem effects, as oil-bound sediment in the northern Gulf of Mexico will likely remain a contamination source for years to come.

## Introduction

The explosion and collapse of the Deepwater Horizon oil platform on April 20, 2010 resulted in the second largest release of oil in history [1]. Millions of barrels of crude oil entered the ecologically and economically important ecosystems in the northern Gulf of Mexico (nGoM) [2]. More than 1773 km of coastline from Louisiana to Florida experienced some form of oil exposure [3]. The application of dispersant [4] was intended to remove much of the oil from the ocean surface which resulted in a large portion of oil sinking to sediments [4,5].

With the release of an unprecedented amount of oil came efforts to examine observed and potential effects on the nGoM ecosystem. Carbon from oil entered the planktonic food web [6] and clear impacts on bacterial communities and processes were demonstrated in subsurface waters [7–9], surface waters [10] and sediments [8,11]. Multiple studies examined oil spill impacts on resident Gulf of Mexico benthic or benthopelagic invertebrates [12–15], planktonic invertebrates [16,17], fish [18–23] and marine mammals [24]. Of particular interest to this study are observations during the oil spill of increased incidences of external lesions or sores on nGoM fish species that are indicative of bacterial infections such as vibriosis [25]. Increased incidence of external lesions has been observed in fish collected from waters containing large concentrations of hydrocarbons [25,26]. Exposure has been tied to depressed immune function and increased susceptibility to pathogen infection in a wide range of fish species [27–36].

In this study, we examined the impacts of oil exposure on immune function and susceptibility to pathogenic bacteria in the southern flounder (*Paralichthys lethostigma*), a benthic-associated fish that supports an important sport fishery in the Gulf of Mexico [37]. Juvenile flounder were exposed to oil-contaminated sediment for 7 days, then challenged with a known fish pathogen, *Vibrio anguillarum* for 1 hour. *V*. *anguillarum* is present in the Gulf of Mexico [38,39] and is the causative agent of vibriosis [40], a hemorrhagic disease responsible for severe economic losses, especially in aquaculture fisheries [40,41]. After pathogen challenge, the flounder were returned to their respective exposure tank, and sampled at 24 hours post-challenge. Our data show that the observed mortality in the combined treatment is a result of oil-induced immunosuppression leading to increased accumulation of pathogenic bacteria, vibriosis, and death.

## Materials and methods

### Organism culture

All exposures were conducted under approval from University of Southern Mississippi (USM) IACUC, number 11092204, specifically for toxicity testing using small fish species. All fish were cultured, exposed, and sacrificed following approved USM IACUC protocol. Juvenile flounder (~90 days post-hatch) obtained from the University of Texas Marine Science Institute were maintained in flow-through tanks and fed brine shrimp nauplii and commercial fish food (Finfish silver, Zeigler® brand) twice per day prior to the experiment. All fish were held in water within 2°C of experimental tanks within 48 hours of experiment.

### Experimental design

The experiment consisted of a fully factorial exposure design with contaminated sediment and pathogen exposure as main factors. There were four treatments: 1) Control (No Oil/No Pathogen challenge), 2) Oil exposure without pathogenic bacteria challenge (Oil/No Pathogen challenge), 3) No oil exposure followed by pathogenic bacteria challenge (No Oil/Pathogen challenge), and 4) oil exposure with pathogenic bacteria challenge (Oil/Pathogen challenge). Oil-contaminated sediment exposures (Oil/No Pathogen challenge and Oil/Pathogen challenge) were performed by mixing uncontaminated field collected sediment (30.37937, -88.30678 collected on 05/15/2013) with a sample of weathered oil ([42] that was collected from the nGoM on July 1, 2010 (Slick B). The concentration of the oil in the artificially contaminated sediment was measured to be 57.4 mg/kg of sediment tPAH50 (the sum of 50 individual PAH concentration measurements). Composite sediment and water samples were analyzed for PAH composition following the procedure discussed in Brown-Peterson et al. (2015) (S2 Table). This concentration was selected as it is environmentally relevant to the Gulf Coast following the oil spill [43] and is a concentration we have previously demonstrated to have adverse effects in flounder [42].

The exposures were performed in triplicate 10-gallon aquaria containing 2 kg/tank of sediment with 20 L of artificial seawater at a salinity of 15, and eight juvenile flounder. Each exposure tank received clean seawater at a rate of 666.7 mL/hour, which maintained optimal water quality parameters (temperature, pH, oxygen, ammonia, salinity). Tanks were monitored twice daily by a trained aquaculture technician for abnormal behaviors or evidence of fish distress (i.e. inverted swimming, gulping for air, rapid uncoordinated movement, or visible lesions), mortality (any dead fish were removed) and water quality parameters. To ensure that no organisms suffered undue stress, any fish observed exhibiting these behaviors was immediately removed and euthanized by immersion in buffered MS-222 until mortality. During the duration of this experiment, no fish were observed exhibiting signs of non-exposure related stress. All water quality parameters remained within nominal standards for the chosen species.

After a seven-day oil exposure, the fish were removed from the exposure tank for a 1-hour bacterial challenge. Flounder assigned to control and Oil/No Pathogen challenge were placed into 10-gallon tanks containing 20 L of bacteria-free filtered seawater. Fish from No Oil/Pathogen challenge and Oil/Pathogen challenge treatments were placed into 10-gallon tanks with 20 L of filtered seawater containing 9.03 X 10^5^ ± 5.09 X 10^4^ cfu/mL *V*. *anguillarum* (ATCC strain 19264). After the 1-hour challenge, the flounder were returned to their respective exposure tanks for 48 hours. At 24 hours post-challenge, two fish were sacrificed from each tank. Liver, spleen, kidney, intestine and gill samples were removed, preserved in RNALater, and stored at -80°C until further processing.

### 16S amplicon sequencing

To examine any alterations in bacterial community structure as a result of oil exposure, samples of oiled and non-oiled tank water and sediment (collected on Day 1 of experimental exposure), as well as upper gill, lower gill and intestine were collected (collected 24 hrs post-pathogen exposure). DNA was extracted from tissues using a PowerSoil DNA Isolation Kit with minor adaptations (MoBio Laboratories). A thoroughly homogenized aliquot of each tissue sample was added to each PowerSoil bead tube. Extraction proceeded per the directions in the kit resulting in 100 uL of DNA in elution buffer (10 mM Tris). Concentrations of DNA in each sample were measured and recorded using a NanoDrop Spectrophotometer (Thermo). The relationship between microbial communities in intestine and gill tissues of oil, pathogen, and co-exposed fish was determined by amplification and sequencing of the V1-V3 variable regions of the gene encoding 16S rRNA [44]. Sequences that failed to return at least half the expected amplicon length (or 250 bp, whichever was shortest) were removed from the data pool. All sequences were then denoised using an algorithm based on USEARCH and checked for chimeras using UCHIME [45]. After denoising and chimera checking, sequence data were separated into operational taxonomic units (OTUs) and annotated using the RDP classifier [46] with GreenGenes v. 12.10 (28) used as a reference. Finally, relative abundances of taxa at each hierarchical taxonomic level were calculated using the summarize taxa.py QIIME script.

Of 1182 OTUs, 699 mapped to known taxonomic assignments (minimum 97% similarity). Those OTUs that failed to match were excluded from further analyses. Negative binomial generalized linear models were performed with the Deseq2 package within R. Treatment effects were statistically analyzed with a Wald test with Benjamini-Hochberg correction. Alpha values for the Benjamini-Hochberg adjusted p-values were set at 0.1. A principal coordinates analysis on a weighted UniFrac metric [47] was calculated to determine if microbial assemblages were different between treatments and to identify which genera were contributing to site differences. Community composition analysis was performed via a Constrained Analysis of Principal Coordinates (CAP).

#### Predictive metagenomic analysis

Closed-reference OTUs were picked from the Green Genes database (v13.5) with QIIME (v1.8.0) at 97% identity [28]. Metagenome predictions were calculated with PiCRUSt (Phylogenetic Investigation of Communities by Reconstruction of Unobserved States, v1.0.0 [23]). From 890 OTUs, 6909 pathways were calculated with 21,940,124 individual predicted gene counts. The average Nearest Sequenced Taxon Index (NSTI) for oiled and non-oiled samples for the metagenomic predictions was 0.14+/- 0.05. Lower NSTI values indicate that microbes in a given sample are more closely related to sequenced genomes [32]. Our NSTI values were consistent with previous work [23], which revealed higher NSTI values in mammalian guts (0.14+0.06) and environmental communities (0.17+0.02). The predicted Kyoto Encyclopedia of Genes (KEGG) pathways were collapsed down to level 3 KEGG Orthology groups (KOs) with the PiCRUSt script categorize by function.py. Negative binomial generalized linear models were fit to the predicted KOs of the microbiota samples. Alpha values for Benjamini-Hochberg adjusted p-values were set at 0.1.

### qPCR

To examine the effect of oil exposure on gene expression, we used quantitative PCR (qPCR) to determine the differential gene expression of several selected genes of interest. To verify that the observed responses were following exposure to hydrocarbons, we quantified gene expression of CYP1A1 in upper and lower gill tissue as a common biomarker of hydrocarbon exposure [48,49]. To assess the effects of exposure to oil-contaminated sediment on immune function of flounder, we also quantified the expression in spleen and kidney tissue of an immune gene (Immunoglobulin γ (IgM)) and one gene important to erythrocyte production and oxygen transport (β-hemoglobin). Total RNA was extracted from tissue samples using either Trizol (Life Technologies, Inc.) or an RNA/DNA Purification Kit (Norgen Bioteck Corp.) and cDNA was constructed using Revertaid First Strand cDNA Construction Kit (Thermo Scientific, Inc.). All qPCR reactions were conducted in triplicate using primers in S1 Table with Fast SYBR Green Master Mix (Life Technologies) on an Applied Biosystems 7500 Fast Cycler. Relative quantification values were determined using the ΔΔC_T_ method in comparison to control samples using amplification of nuclear ribosomal 18S as an internal reference.

### *De Novo* transcriptome assembly

To obtain a genome-wide view of the flounder transcriptome and gene expression profile following oil exposure, high-throughput RNA-seq was performed using Illumina sequencing technology. RNA was isolated from livers as discussed above and sequenced on an Illumina HiSeq2000. Paired-end sequence reads from all libraries (S1 Table) were pooled to generate a *de novo* transcriptome assembly for the southern flounder (Workflow described in S1 Fig). Singletons sequence reads assembled into unitigs with ABySS v. 1.3.7, using 11 unique k—mers between k = 40 and k = 90 in increments of 5. ABySS [50] was run with default parameters except requiring a minimum k—mer coverage of 5, graph bubble popping at >0.9 branch identity, with the scaffolding flag disabled to avoid over reduction of divergent regions. Unitigs from all k—mer assemblies were combined and redundancies removed using CD-HIT-EST [51] with a clustering threshold of 0.98 identity. The OLC (Overlap-Layout-Consensus) assembler CAP3 [52] was used to identify minimum 100 bp overlaps between the resultant unitigs to assemble larger sequences. Extended sequences from CAP3 were paired-end scaffolded using ABySS [50]. Sequence read pairing information was used in GapCloser {[53], part of SOAP *de novo* package} v. 1.12 to walk in gaps created during assembly. To remove incomplete sequences, the consensus scaffolds were filtered at a minimum length of 200 bp to produce the final set of scaffolds. The final assembly contained 65,947 synthetic ESTs with a scaffold N50 1,500 bp. See S2 Table for additional transcriptome assembly metrics generated using GAEMR (http://www.broadinstitute.org/software/gaemr/) from the Broad Institute.

#### Differential expression analysis

Following *de novo* assembly of the Southern flounder (*Paralichthys lethostigma*) transcriptome, differential transcript expression analysis was performed pairwise for each of the four treatment groups. Each treatment group contained three replicates. Raw fastq files were quality checked using the FastQC program, version 0.10.0 [54] and mapped/aligned to the Southern flounder *de novo* transcriptome using Bowtie2, version 2.2.1 [55]. Bowtie2 was run in paired-end mode with the minimum fragment length (-I) and maximum fragment length (-X) parameters set to 50 and 300 nucleotides, respectively. The seed length (-L) and the number of mismatches permitted per seed (–N) options were set to 25 and 1, respectively. After mapping the reads to the transcriptome, the mapped/aligned reads in BAM format, were quantified using the RSEM algorithm, version 1.2.19 [56]. RSEM was run in paired-end (—paired-end) mode with default parameters. The read counts generated by RSEM were utilized to perform differential transcript expression analyses using the EBSeq algorithm, version 1.4.0 [57] with default parameters. Differentially expressed transcripts were filtered by a posterior probability of differential expression (PPDE) of greater than or equal to 0.95. Transcripts with a PPDE > = 0.95 are differentially expressed transcripts with a target false discovery rate (FDR) controlled at 5% (FDR < = 0.05). Significantly, differentially expressed transcripts were used to create a hierarchical cluster and heatmap in *Cluster3*.*0* and *Treeview*.

#### Gene/Pathway enrichment analysis

The filtered lists of differentially expressed transcripts for each pairwise comparison (PPDE > = 0.95) were annotated with uniref100 ids that served as transcript identifiers. The uniref100 ids were converted to Ensembl transcript ids or associated gene names using the UniprotKB database [58]. The Ensembl transcript ids and/or associated gene symbols were then converted to orthologous *Danio rerio* (Zebra fish) Ensembl gene ids using the Ensembl biomart database [59]. The *Danio rerio* Ensembl gene ids obtained from Ensembl biomart were used to perform gene/pathway enrichment analyses using the ClueGo app, version 2.1.7 [60] and Cytoscape version 3.2.1 [61].

Enrichment analyses, using ClueGo, were performed separately for up-regulated or downregulated transcripts for each pairwise comparison. ClueGo was run using *Danio rerio* as the target model species. The GO:Biological Process and Reactome or GO:Molecular Function database options were selected from the “Ontologies/Pathways” menu. The “Show only pathways with p-value” option was set to 0.05, meaning that enriched genes/pathways were only reported if they had an adjusted p-value < = 0.05. The remaining options were set to defaults. Using annotated transcriptomes, putative gene products were assigned to major gene ontology (GO) categories and those associated with enriched GO terms were grouped into biological processes and molecular function categories. RNA sequencing data is publicly available in NCBI’s SRA database under the following accession numbers; SRR3737288, SRR3738769, SRR3744019, SRR3744655.

## Results and discussion

**Fig 1:**
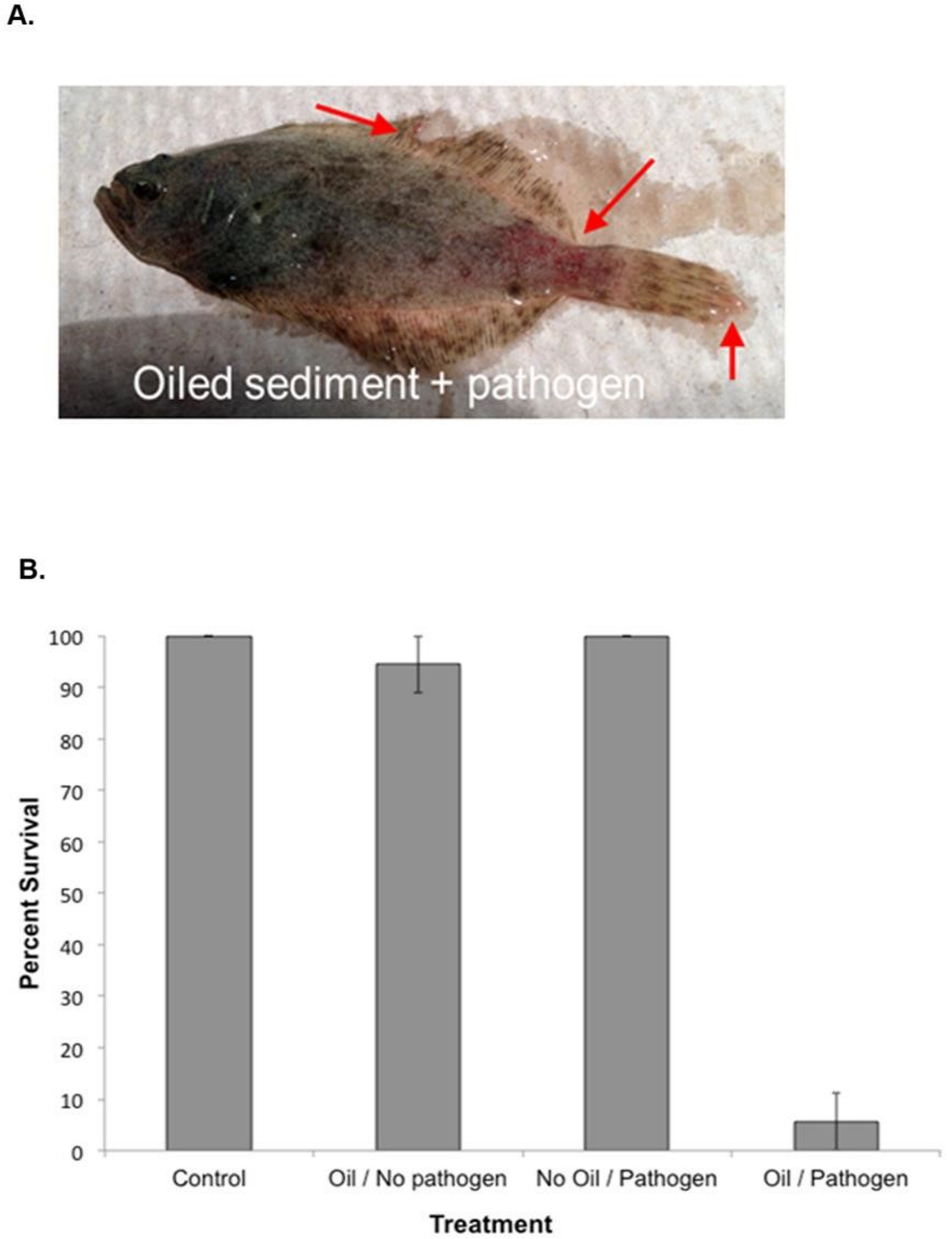
**(A) Evidence of bloody lesions seen in flounder exposed to oil.** Red arrows indicate observed lesions. Fish were not tagged; therefore, lesions were not enumerated. **(B) Survival rates in exposure treatments on Day 2 post-exposure to pathogenic bacteria *Vibrio anguillarum***. Bars represent average survival for three tanks per treatment and error bars represent standard error.

Increased observations of lesions in multiple fish species sampled post-DWH suggested, but could not definitively link, oil exposure to lesion prevalence [25]. In this controlled laboratory-based study, juvenile flounder exposed to oil experienced visible bloody lesions similar to those reported in the field (Fig 1A). Furthermore, fish in several oil exposed treatment tanks were observed swimming to the surface and gulping for air, even though oxygen levels were adequate at those times (6.46 ± 0.16 mg/L). Fish from non-oil tanks did not exhibit these behaviors or lesions. Ultimately, flounder mortality was significantly elevated (94.4%) in Oil/Pathogen challenged treatments at 48h post challenge (Fig 1B). Mortality in all other treatments was less than 10%.

**Fig 2:**
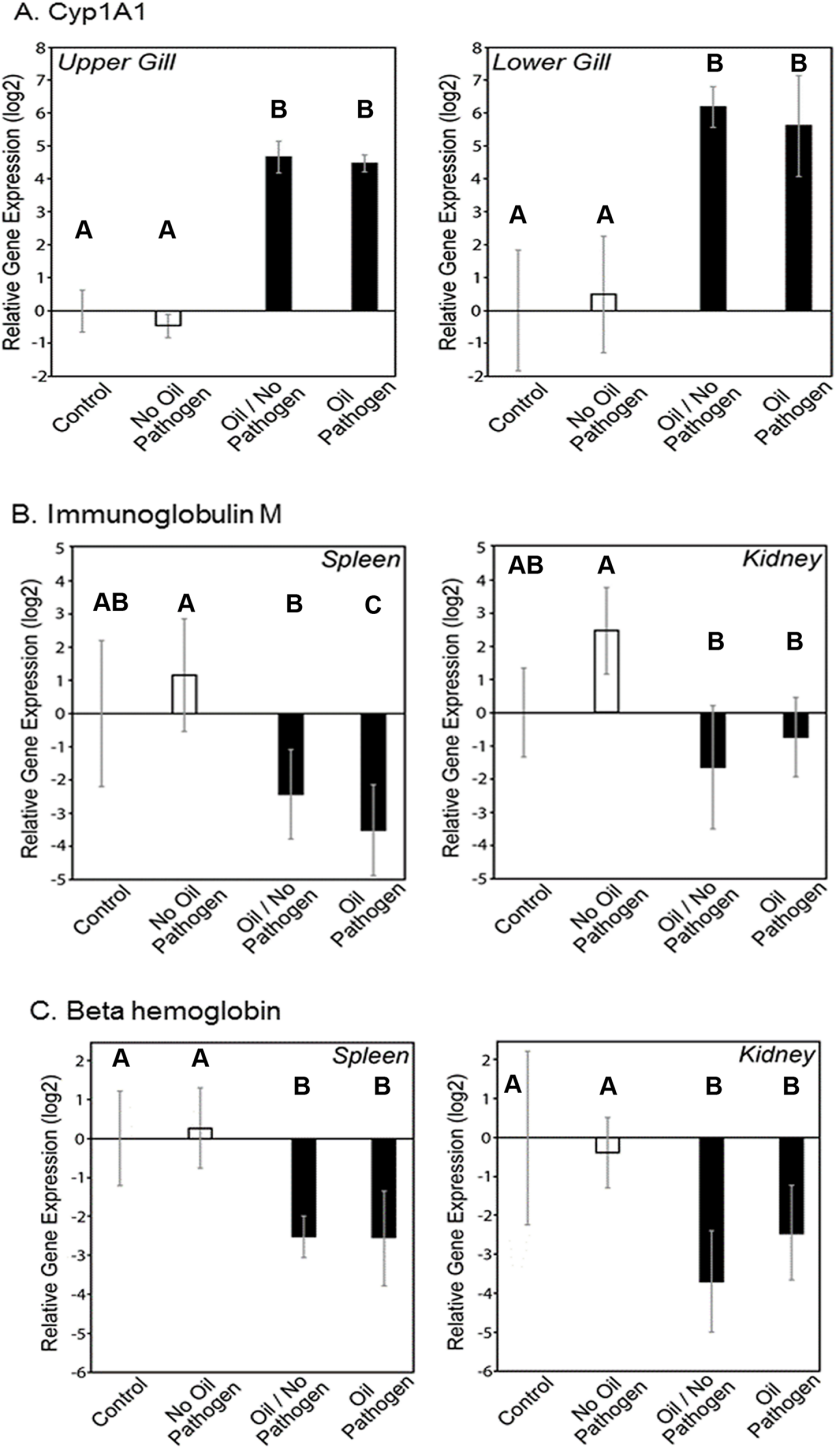
**Effect of oil exposure on relative gene expression for A) CYP1A in upper and lower gill tissue and B) Immunoglobulin M and C) Beta hemoglobin in spleen and kidney tissue of fish collected Day 1 post-pathogen exposure.** Bars represent average gene expression (log_2_) relative to control fish for 3 fish per treatment and error bars represent minimum and maximum values of relative quantification based on standard deviation of Ct values from qPCR analyses.

Oil exposure strongly up-regulated expression of cytochrome P-4501A (CYP1A1) in both upper and lower gill tissue (Fig 2A). Elevated expression of CYP1A is indicative of hydrocarbon metabolism via the Phase I biotransformation pathway, and is a commonly used biomarker for exposure to oil [48,49]. Increased CYP1A1 mRNA expression indicated oil exposure in marsh fish collected post-spill in the nGoM [23,62]. The fact that lower gill tissue exhibited a higher abundance of CYP1A mRNA than upper gill tissue directly links oil contamination to lower gill tissue exposure.

Oil exposure resulted in down-regulation of β-hemoglobin gene expression (Fig 2C), a gene important for erythrocyte production and oxygen transport. A lowered hemoglobin concentration has been linked to oil exposure. Winter flounder (*Pleuronectes americanus*) collected from waters with elevated PAH levels exhibited decreased concentrations of hemoglobin compared to reference animals [26]. European flounder (*Pleuronectes flesus*) exposed to the water-soluble fraction of crude oil exhibited decreased levels of hematocrit and hemoglobin [63]. Down-regulation of hemoglobin genes (including β-hemoglobin) has been demonstrated for *Paralichthys olivaceous* [36,64] and *Oncorhyncus mykiss* [65,66] in response to exposure to various oil types (heavy, diesel, kerosene, etc.) and individual PAHs. β-hemoglobin down-regulation resulted in lowered erythrocyte levels, leading to a reduction in oxygen transport [36,64,66], which was observed when the cladoceran *Daphnia magna* was exposed to naphthalene [67]. A reduction in oxygen transport driven by lowered hemoglobin expression was used to explain the behavior of oil-exposed *O*. *mykiss* “gulping” for air at the surface when oxygen levels were high [66]. We observed similar instances of oil-exposed flounder swimming to the surface in well-oxygenated tanks. The resulting down-regulation in β-hemoglobin later recorded in fish from those tanks further suggests a highly negative impact of oil to fish health.

Oil exposure also resulted in reduced expression of IgM mRNA, the primary systemic fish antibody and typically one of the first to respond to bacterial infection [68–70]. Expression of IgM was reduced in both spleen and kidney tissue from flounder exposed to oil-contaminated sediment (Fig 2B). IgM is one of three antibodies formed in most fish species [71,72] and it is the most prominent and most prevalent systemic fish antibody [69,70]. Exposure to heavy oil resulted in significant down-regulation in IgM in both spleen and kidney for *P*. *olivaceous* [36,64] and it is suggested that exposure to the PAH benzo[a]pyrene decreases antibody-forming cell numbers in exposed fish [73], via CYP1A-catalyzed production of PAH metabolites [74]. While benzo[a]pyrene is not present in high concentrations in Deepwater Horizon oil [75], it is possible that similar mechanisms with a different suite of hydrocarbons may explain the down-regulation in IgM, given the up-regulation in CYP1A expression seen in this study.

**Fig 3:**
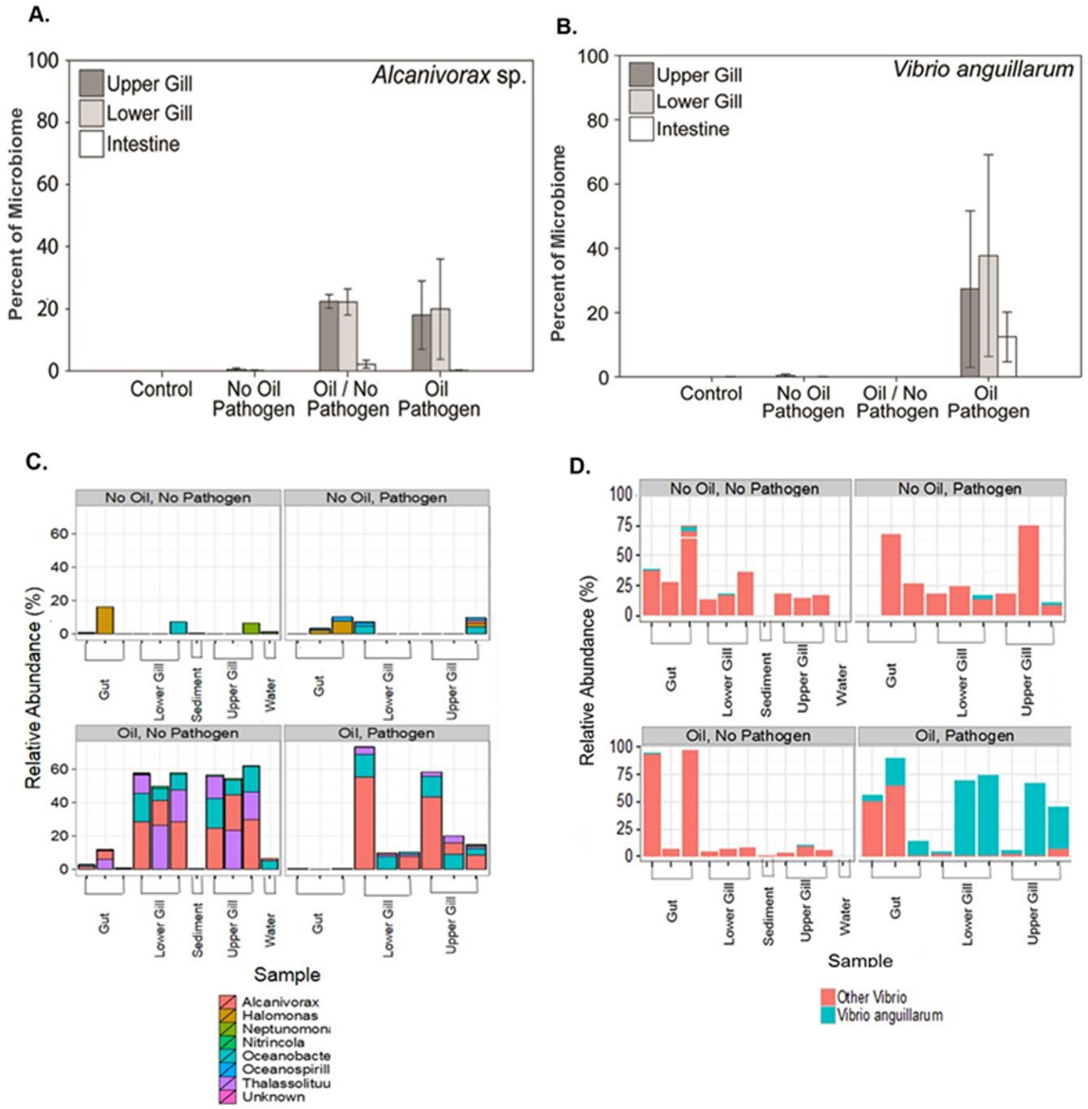
Microbial communities significantly affected by oil exposure. **(A)** Comparison of the percent of Alcanivorax with **(B)** the experimental pathogen *Vibrio anguillarum* in microbiome found in the upper gill, lower gill and intestine from flounder tissue collected Day 1 post-pathogen challenge (3 fish/treatment). Mean percentage of microbiome (+/- 1 SEM) for *Alcanivorax* sp. and *V*. *anguillarum*. (C) Comparison of the relative abundance of PAH degrading bacterial groups such as Alcanivorax, Halomonas and Oceanospirillales in the gut, lower gill, upper gill, sediment and water collected Day 1 post-pathogen challenge after flounder were returned to experimental tanks and **(D)** a closer investigation comparing the relative abundance (%) of the bacterial challenge species *Vibrio anguillarum and other Vibrio* spp between water, sediment and experimental treatments sampled Day 1 post-pathogen challenge.

One of the most striking observations was that flounder exposed to oil were less effective at defending against pathogen infection due in part to IgM down-regulation. *Alcanivorax sp*. (Fig 3A) and *V*. *anguillarum* (Fig 3B) comprised noticeable portions of the microbiome in Oil/Pathogen challenged fish compared to No Oil/Pathogen challenged fish. The relative abundance of oil degrading bacteria, including Oceanospirillales, Thalassolituus and Alcanivorax, comprised up to 60% of the microbial community in the lower and upper gills of fish exposed to oil (Fig 3C). Additionally, the relative abundance of *V*. *anguillarum* were negligible in No Oil/Pathogen challenged fish but comprised up to 75% of the lower and upper gills of Oil/Pathogen challenged fish indicating a immunocompromisation induced by oil exposure (3D). In several fish species, a reduced ability to defend against pathogen infection occurred after exposure to oil or its components [27,29,35,36,64,76,77]. Rainbow trout experienced a 10% higher mortality following a 50 dy dietary PAH exposure and disease challenge as compared to control fish [76]. Similarly, sea bass challenged with a Viral Nervous Necrosis Virus experienced elevated mortality when previously exposed to dispersed oil, however, there were no lasting impacts on the innate immunity [77]. In the congener species *P*. *olivaceous*, exposure to heavy oil resulted in higher concentrations of bacteria [34] and lowered antibacterial activity [36] in skin mucus for oil-exposed fish when compared to unexposed fish. In addition, exposure to heavy oil increased mortality due to viral infection in *P*. *olivaceous* [35]. The fact that IgM down-regulation was only observed in oil-exposed flounder, that increased colonization *by V*. *anguillarum* only occurred in fish with down-regulated IgM and that mortality only occurred in fish with down-regulated IgM and increased *V*. *anguillarum* produces a clear mechanism for mortality.

**Fig 4:**
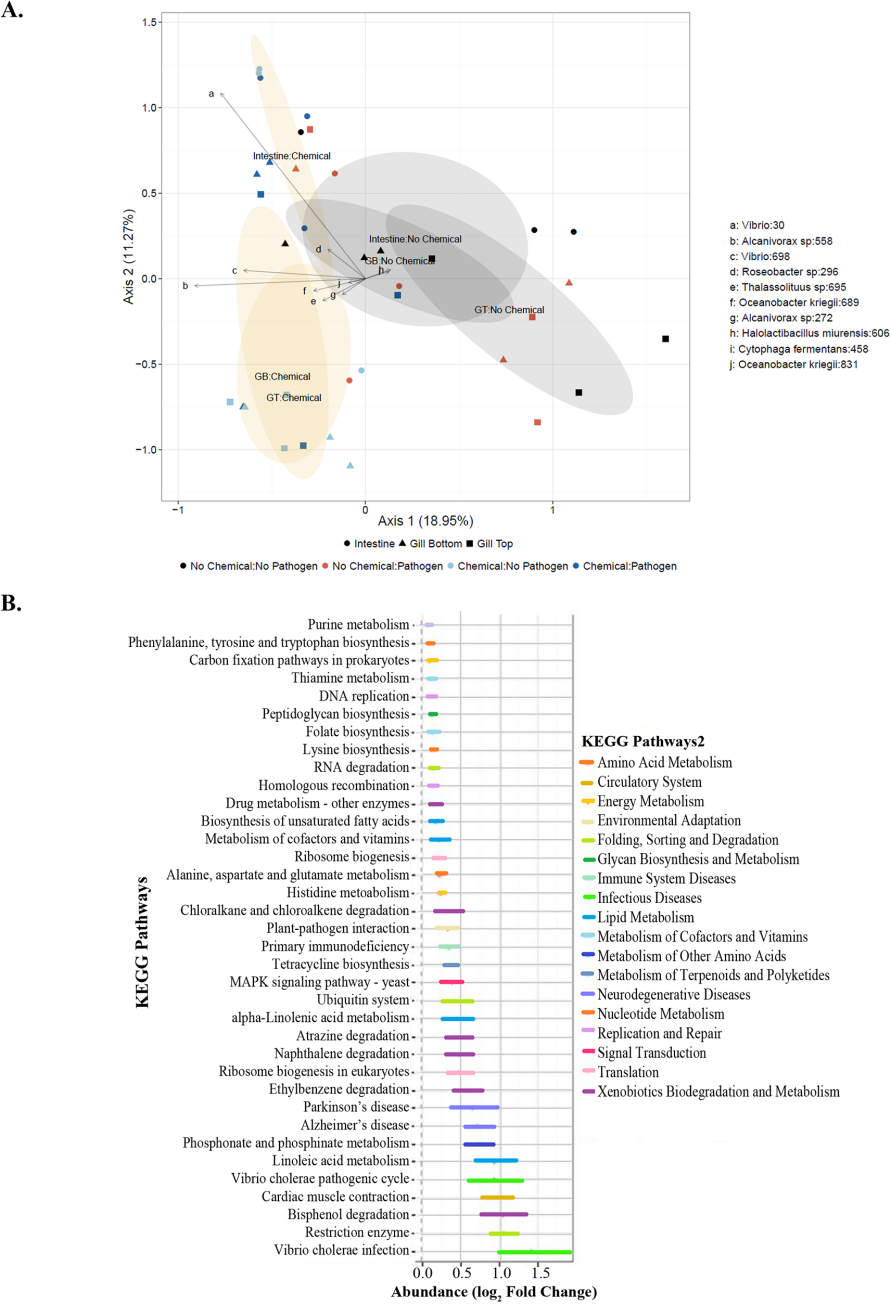
Effect of oil exposure on bacterial diversity and function. **(A)** Principal Coordinates Analysis (PCoA) plot of data at the Order level from fish collected on Day 1 post-pathogen exposure calculated with a Bray-Curtis dissimilarity in R (v. 3.0.2 [2013-09-25]–“Frisbee Sailing”). Water and sediment samples were collected on Day 0 of the experiment. Lower case letters indicate locations of various bacterial orders (a-*Vibrio*, b-*Alcanivorax* sp, c-*Vibrio*, d*Roseobacter*, e-*Thalassolitus*, f-*Oceanobacter kriegii*, g-*Alcanivorax* sp, h-*Halolactibacillus miurensis*, i-*Cytophaga fermentans*, j-*Oceanobacter kriegii*). Symbols represent different tissue types (● Intestine, ▲Lower Gill, ■ Upper Gill). Ellipses represent non-oil exposed vs. oilexposed each defined by treatment labels in their centers and indicate 95% Confidence Intervals, with orange ellipses representing both oiled treatments and gray ellipses representing both nonoiled treatments. **(B)** Predictive metagenomics analysis of the intestinal bacterial taxa in Oil/Pathogen Challenged fish (compared with control fish) indicated enrichment in degradation pathways. Pathway abundance was normalized via log_2_ fold change.

In addition, oil exposure resulted in consistent and clear shifts in overall bacterial community composition in gills and intestines of fish collected one day after pathogen challenge (Figs 3 and 4). Principal coordinates analysis of data collected from all organs for 12 fish (3 per treatment) indicated that oil-exposed and non-oil-exposed fish contained taxonomically distinct bacterial communities, with gill and intestinal communities from oiled fish also differing from each other (Fig 4A). For example, the hydrocarbon-metabolizing bacteria *Alcanivorax* sp. was only found in fish exposed to oiled sediment (Fig 3A). Bacteria of the genus *Alcanivorax* are key alkane degraders in marine sediments [78,79]. *Alcanivorax* was found in increased quantities in marine sediments [8,11] and seawater [8,80,81] impacted by Deepwater Horizon oil and it has been demonstrated that, when supplied with adequate nutrients (nitrogen and phosphorus), it will become the predominant bacterial species in seawater containing petroleum. Consistent with these attributes, *Alcanivorax* and many other PAH-degrading groups increased in abundance in the sediment and gills adjacent to the sediment (Fig 3C). Furthermore, predictive metagenomic analysis utilizing PICRUSt algorithms indicated the up-regulation of pathways in Oil/Pathogen challenged intestines associated with *Vibrio* infection, *Vibrio* pathogenic cycles, bisphenol degradation, and other organic molecule degradation, which are indicative of both oil degradation and *Vibrio* pathogenesis (Fig 4B).

Such significant shifts in gill and intestinal bacterial composition would have significant detrimental systemic effects on oil-exposed fish. Communities of commensal bacteria, especially in the gastrointestinal system, are important for organismal homeostasis. Imbalances in these communities can have detrimental physiological effects [82]. The intestinal microbiome is important for proper nutrient metabolism and absorption, gut epithelium development and defense, energy balance and immunity [82]. Nutritionally, the gut microbiome produces amino acids, vitamins, and enzymes necessary for efficient digestion [83,84]. Additionally, the intestinal microbiome plays an important role in the development of the gut immune system, or the gut-associated lymphoid tissues, which act as a physical defense against pathogens, in addition to regulating the immune response in the alimentary canal [82,85,86]. An imbalance or shift in gut microbial communities can result in the successful establishment of lethal infections by pathogenic organisms [87]. Given the importance of organismal microflora in numerous physiological, nutritional and immune processes, the observed shifts in the community composition (Figs 3 and 4) could result in similar detrimental effects to these processes in oil-exposed fish.

**Fig 5:**
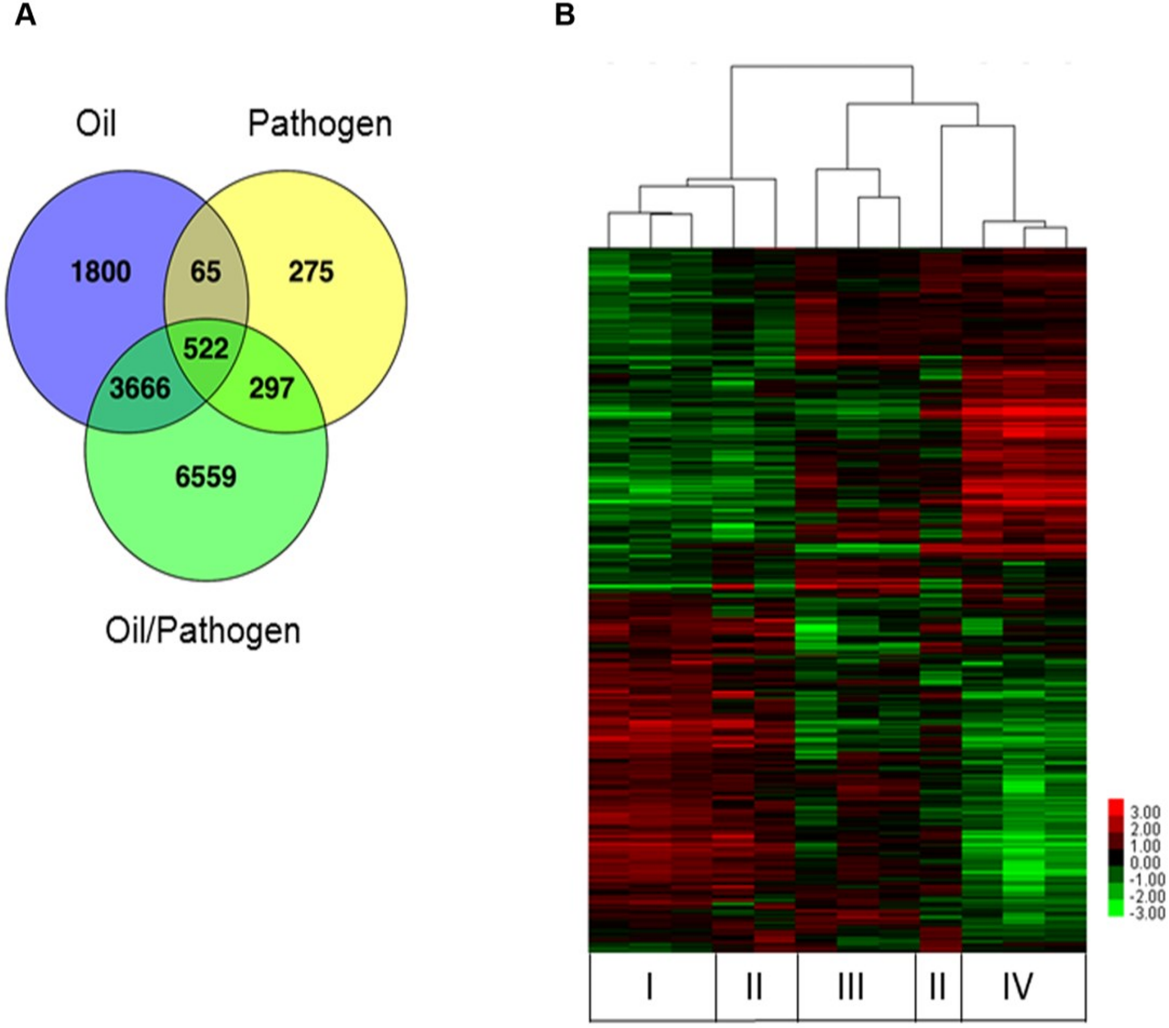
Differentially expressed transcripts in livers of flounder exposed to oil. **(A)** The Venn diagram illustrates the comparison of each treatment group with the control group based on the significantly differentially expressed genes (n = 13184 response genes). **(B)** The hierarchically clustered transcript heatmap was generated using average linking distance metric of the log_2_ transformed (FPKM) values. Green represents lower expression, red represents high expression compared to the controls, columns represent individual experiments (No Oil/No Pathogen (I), Pathogen/No Oil (II), Oil/No Pathogen (III), Oil/Pathogen (IV)), and rows represent transcriptional units.

RNA-seq results revealed significant effects on multiple biological processes, further supporting the evidence for oil exposure negatively influencing the ability of flounder to properly fight infection and regulate normal cellular activity, as was evident in the effect on the microbiome (Fig 3A and 3D). While the liver is not the primary site of fish immune regulation, genes important in immune system pathways were significantly suppressed suggesting that there is a suite of liver immune genes responding to contamination. GO enrichment indicated that many biological processes were suppressed by oil exposure, including immune function, xenobiotic response and stress response. The comparison between the transcriptomes of control and all treatment groups revealed 13,184 differentially expressed transcripts. 522 differentially expressed transcripts were shared between all treatments. 3666 transcripts were shared between Oil/Pathogen challenge and Oil/No Pathogen challenge treatments. Few transcripts (n = 65) were shared between Oil/No Pathogen challenge and No Oil/Pathogen challenge, suggesting that oil exposure causes an entirely different transcriptional response than pathogen exposure. The greatest difference in transcription level (n = 6559) occurred between control and Oil/Pathogen challenged fish, and other comparisons showed much less effect. The difference between control and Oil/No Pathogen was less dramatic, and very little transcriptional difference occurred between control and No Oil/Pathogen fish (Fig 5A). Hierarchical clustering clearly illustrated a universal difference in transcriptome expression when flounder are exposed to oil versus pathogen challenge alone (Fig 5B). Ultimately, the concentration of oil used did not detrimentally affect the fish, however, the addition of the bacterial challenge became too much for them to overcome. It is possible that some of the global transcriptional alterations observed in the Oil/Pathogen treatment are due to incipient mortality as all, but one of the remaining fish died within 24 hours of the sampling event (by 48h post-challenge). However, as the fish appeared largely healthy at the time of the sampling, and mortality did not commence for at least 12 hours post-sampling, we do not believe this is a driving factor in the transcriptional data.

Multiple lines of evidence point to oil-exposed pathogen challenged flounder fighting infection and metabolizing toxins simultaneously. For example, oil-exposed flounder challenged with *V*. *anguillarum* experienced reduced expression of genes involved in the pathways for xenobiotic metabolic processes (*cyp2ad2*, *cyp2x6-9*, *gadph*, and *hao1*) and Phase-I functionalization of compounds (*mgst1*, *tpmt*, *acy1* and *sult*), compared to control treated animals. The dysfunction of these important chemical defense pathways in the liver suggests flounder struggle with fighting *V*. *anguillarum* infection and metabolizing toxins simultaneously, which allows for the increased colonization of *V*. *anguillarum* in fish exposed to oil as compared with pathogen challenge only (Fig 3B). Increased aerobic respiration expression (*mtfr1*, *uqcrc1*) in conjunction with observed gulping and the downregulation of β-hemoglobin supports severely stressed juvenile flounder. Furthermore, gut microbial activity contributes to the maintenance of the host’s cellular homeostasis, including vitamin production and the shift in that microbial community seen in juvenile flounder may alter the ability to synthesize vitamins. Oil/Pathogen challenged fish increased expression of genes involved in the metabolism of vitamins and cofactors pathway (*coasy*, *pank1*, *tpk1*) possibly mediating the loss of important vitamin synthesizing microbes as seen in the shift of relative abundance of the microbial community in the intestines of flounder (Fig 3B). Deficiencies in vitamin A, for instance, results in increased susceptibility to pathogen infection in mice [88]. B-group vitamin members are involved in essential cellular functions including DNA replication, repair and methylation and amino acid synthesis [89,90]. The combination of vitamin metabolism activation, oxygen-stress and dysfunction of the chemical defense system further supports a mechanism for mortality of Oil exposed/Pathogen challenged flounder [89,91].

Oil/No Pathogen challenge livers featured significantly suppressed genes related to hemostasis (*c-srctyrosine kinase* and *syk*), the regulation of immune systems (*apom*, *irf8*, *spi1b* and *gata3*) and the regulation of response to stimulus (*k1h16*, *masp1*, *ptk2b*, *ptprc* and *skap1*) (Fig 5C). These genes included well-known transcriptional targets of the Interferon regulatory factor 8 (*irfa*), which plays a negative regulatory role in cells of the immune system, and the protein coding Spi-1 Proto-Oncogene (*spi1b*) gene which is implicated in the differentiation of β-cells [89,92]. Previous studies examining the acute transcriptomic impact of oil exposure in *P*. *olivaceous* gills found increased expression of mechanisms important to minimizing the toxicity of oil exposure [93]. As expected, in the current study, fish exposed to oil up-regulated specific genes necessary to ameliorate oil contamination, including those involved in DNA repair, oxidative damage response [*pnkp* (polynucleotide kinase 3phosphatase)], suppression of cell dysfunction induced by oxidative stress [*nudt1* (nucleoside diphosphate linked moiety)], and importantly genes involved in the chemical defense pathway (*cyp46a1*). The activation of genes associated with pathways involved in the metabolism of xenobiotic compounds is a typical response to oil exposure in many organisms [92,94].

Juvenile flounder exposed to bacteria alone had similar transcriptional profiles to control fish with the exception of changes to the immune responses. For example, genes involved in homeostasis and amino acid activation were significantly enriched compared to controls while erythrocyte homeostasis and tryptophan catabolism were suppressed. Erythropoiten (EPO), an important regulator of red blood cell production, was significantly expressed which is consistent with our observation that No Oil/Pathogen challenged juvenile flounder are successfully fighting off infection [95].

The sub-lethal effects on fish immunity and bacterial flora driven by oil exposure are also important. Oil/No Pathogen challenged fish showed similar shifts in bacterial flora (Fig 3C) and suppression in IgM expression (Fig 2B) to Oil/Pathogen challenged fish, indicating multiple mechanisms by which oil exposure can affect homeostasis and immune response in oil exposed fish. In natural waters, fish exposed to oil in the sediment are likely more susceptible to infection by pathogenic bacteria due to immunotoxic effects of oil. When oil exposure drives shifts in an organism’s microflora, it can have an overall system-wide impact on nutritional efficiency and nutrient uptake, while also impacting immune response in the gut [82,96].

### Conclusions

The near complete mortality in Oil/Pathogen challenged fish within 48 hrs is linked to the interactions of two processes: the inability to fight off *V*. *anguillarum* infection and the inability to withstand the hemolytic effects of *V*. *anguillarum* by producing more red blood cells. *V*. *anguillarum* was only found in tissues of Oil/Pathogen challenged fish (Fig 3D), fish that also showed down-regulation in immune related genes and β-hemoglobin (Fig 2B and 2C). Similar results have been observed in other hydrocarbon-exposed fish species [26,63–66,97], with down-regulation interpreted as lowered erythrocyte levels leading to a reduction in oxygen transport [36,64,66,67]. This is consistent with our observations of Oil/Pathogen challenged fish “gulping” for oxygen. Conversely, unexposed fish showed no evidence of impaired immune function (Figs 2B and 3D) and unchanged or increased expression in genes related to blood production, such as β-hemoglobin and Erythropoiten (EPO). Our results suggest that the lesions observed on nGOM fish after the DWH oil spill are the result of an immunotoxic response to oil exposure, resulting in an increased prevalence of pathogenic infections.

## Supporting information

S1 Table Primers used in this study. IgM: Immunoglobulin M; HBB: Hemoglobin subunit beta; CYP1A: Cytochrome P-4501A; 18S: Nuclear ribosomal 18S subunit (internal 848 reference).
(DOCX)

S2 Table Amount of sediment, loading rate of Slick B oil, initial and final tPAH50 concentrations, and percent total organic carbon (TOC) of treatments. ǂ Initial measurements taken one day prior to experiment initiation; final measurements taken at experiment termination after 15-d with clean water flow-through.
* Samples for TOC measurements taken during original field collection.
(DOCX)

S3 Table Basic transcriptome assembly metrics. (DOCX)

S4 Table List of differentially expressed transcripts between treatments. * Differentially expressed transcripts filtered by fold change ≥ 1.5 filtered by a PPDE ≥ 0.95.
* Up-regulated genes (realFC ≥ 1.5) or down-regulated (realFC ≤ 0.667 or negative 1.5 fold).
(XLS)

S1 Fig Transcriptome assembly workflow. A) Data pooled from all seven samples. B) Performed k mer sweep setting for 40 through 90, increments of 5, k = (40, 45, 50, 55, 60, 65, 70, 75, 80, 85, 90). Ran ABySS with different k values on the pooled data as single end reads and generate Unitigs. C) Merged Unitigs from all k mers. D) Removed duplicates from the merged set using CD-HIT setting identity threshold at 0.98 (98%). E) Assembled unique unitigs using CAP3 to extend unitigs to larger sequences. F) Performed scaffolding using the scaffolding module (abyss-scaffold) from ABySS using the Paired-End information. G) Attempted to resolve N spacers introduced during the scaffolding process using GapCloser tool H) from the SOAPdenovo suite. I) Removed duplicates again using CD-HIT setting identity threshold at 0.98 (98%). J) Generated final transcriptome assembly.
(DOCX)
