# Supplementary material for: Crude oil impairs immune function and increases susceptibility to pathogenic bacteria in southern flounder
Source: PLoS One. 2017 May 2;12(5):e0176559. doi: 10.1371/journal.pone.0176559 (PMC5413019; doi:10.1371/journal.pone.0176559)
Supplement: S1 Table — IgM: Immunoglobulin M; HBB: Hemoglobin subunit beta; CYP1A: Cytochrome P-4501A; 18S: Nuclear ribosomal 18S subunit (internal 848 reference). (DOCX) [file pone.0176559.s001.docx]

**Supplemental Material**

**S1 Table.**

| Target | Primer Sequence (5’-3’) | Amplification  Efficiency | Amplicon  Size (bp) | Source |
| --- | --- | --- | --- | --- |
|  |  |  |  |  |
| *IgM* | F: TCAGGCTGAAGATGTTGGAGA | 97.88% | 151 | (Song et al. 2012) |
|  | R: AGAGAGCTGCTCAGAGGAAG | - | - | (Song et al. 2012) |
| *Β-hemo* | F: CTTGCTGAGTGCATCAGTGT | 84.91% | 122 | (Song et al. 2012) |
|  | R: TACTGTTTGCCCAGAGCAGA | - | - | (Song et al. 2012) |
| *CYP1A* | F: GTTCGATACCGTCTCTACTG | 88.92% | 345 | (George et al. 2004) |
|  | R: AGGAAGCGATCTGGGTTGAAG | - | - | (George et al. 2004) |
| *18S* | F: GACTCAACACGGGAAACCTC | 90.49% | 110 | (Song et al. 2012) |
|  | R: AGACAAATCGCTCCACCAAC | - | - | (Song et al. 2012) |
