# Supplementary material for: Crude oil impairs immune function and increases susceptibility to pathogenic bacteria in southern flounder
Source: PLoS One. 2017 May 2;12(5):e0176559. doi: 10.1371/journal.pone.0176559 (PMC5413019; doi:10.1371/journal.pone.0176559)
Supplement: S2 Table — ǂ Initial measurements taken one day prior to experiment initiation; final measurements taken at experiment termination after 15-d with clean water flow-through. * Samples for TOC measurements taken during original field collection. (DOCX) [file pone.0176559.s002.docx]

**Supplemental Material**

| **S2 Table**. | | | | |  |
| --- | --- | --- | --- | --- | --- |
|  |  |  |  |  |  |
|  |  |  |  |  |  |
|  |  |  |  |  |  |
| Treatment | Sediment mass (kg) | Nominal loading concentration (g/kg) | Measured concentration at initiation  (mg/kg tPAH50) | Measured concentration at termination (mg/kg tPAH50) | TOC (%)* |
| Control | 18 | NA | 0.020 | 0.022 | 0.434 |
| Oil exposed | 18 | 27 | 57.432 | 37.552 | 0.434 |
|  |  |  |  |  |  |
|  |  |  | Measured concentration at initiation  (µg/L tPAH50) | Measured concentration at termination (µg/L tPAH50) |  |
| Unfiltered Water Control |  |  | 0.053 | 0.000 |  |
| Unfiltered Water Oil Exposed |  |  | 47.958 | 3.546 |  |
| Filtered Water Control |  |  | 0.044 | 0.114 |  |
| Filtered Water Oil Exposed |  |  | 0.475 | 0.509 |  |
|  |  |  |  |  |  |
| ǂ Initial measurements taken one day prior to experiment initiation; final measurements taken at experiment termination after 15-d with clean water flow-through.  * Samples for TOC measurements taken during original field collection. | | | | |  |
