# Supplementary material for: Crude oil impairs immune function and increases susceptibility to pathogenic bacteria in southern flounder
Source: PLoS One. 2017 May 2;12(5):e0176559. doi: 10.1371/journal.pone.0176559 (PMC5413019; doi:10.1371/journal.pone.0176559)
Supplement: S3 Table — (DOCX) [file pone.0176559.s003.docx]

**Supplemental Material**

**S3 Table:**

|  | #TITLE: Basic Assembly Stats | |
| --- | --- | --- |
|  | #Name | Flounder |
|  | Assembler | ABySS |
| Contigs | Contigs | 204,704 |
|  | Max Contig | 23,859 |
|  | Mean Contig | 646 |
|  | Contig N50 | 891 |
|  | Contig N90 | 278 |
|  | Total Contig Length | 132,334,478 |
| Assembly GC | | 45.99 |
| Scaffolds | Scaffolds | 160,706 |
|  | Max Scaffold | 24,358 |
|  | Mean Scaffold | 827 |
|  | Scaffold N50 | 1,450 |
|  | Scaffold N90 | 306 |
|  | Total Scaffold Length | 132,909,940 |
| Gap | Captured Gaps | 43,998 |
|  | Max Gap | 2,818 |
|  | Mean Gap | 13 |
|  | Gap N50 | 44 |
|  | Total Gap Length | 575,462 |
|  |  |  |
