# Supplementary figures and images for: Crude oil impairs immune function and increases susceptibility to pathogenic bacteria in southern flounder
Source: PLoS One. 2017 May 2;12(5):e0176559. doi: 10.1371/journal.pone.0176559 (PMC5413019; doi:10.1371/journal.pone.0176559)

**Supplemental Material**

**S1 Figure.**

**
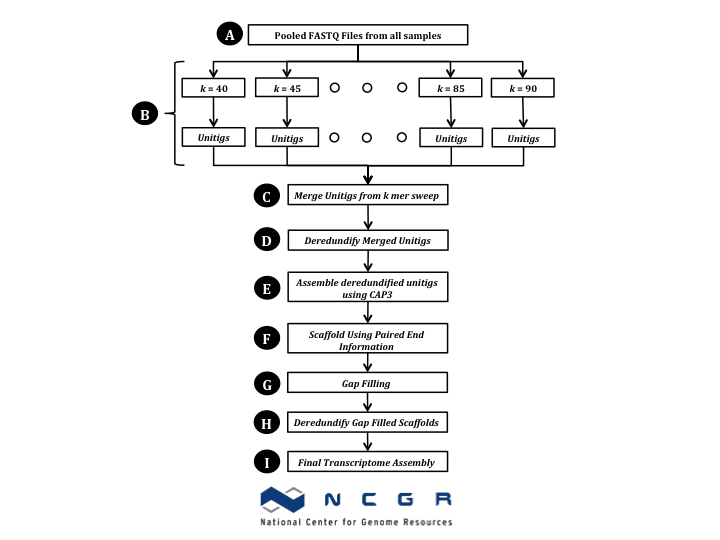
**

Supplement: S1 Fig — A) Data pooled from all seven samples. B) Performed k mer sweep setting for 40 through 90, increments of 5, k = (40, 45, 50, 55, 60, 65, 70, 75, 80, 85, 90). Ran ABySS with different k values on the pooled data as single end reads and generate Unitigs. C) Merged Unitigs from all k mers. D) Removed duplicates from the merged set using CD-HIT setting identity threshold at 0.98 (98%). E) Assembled unique unitigs using CAP3 to extend unitigs to larger sequences. F) Performed scaffolding using the scaffolding module (abyss-scaffold) from ABySS using the Paired-End information. G) Attempted to resolve N spacers introduced during the scaffolding process using GapCloser tool H) from the SOAPdenovo suite. I) Removed duplicates again using CD-HIT setting identity threshold at 0.98 (98%). J) Generated final transcriptome assembly. (DOCX) [file pone.0176559.s005.docx]
